# A zeta potential value determines the aggregate’s size of penta-substituted [60]fullerene derivatives in aqueous suspension whereas positive charge is required for toxicity against bacterial cells

**DOI:** 10.1186/s12951-015-0112-6

**Published:** 2015-08-08

**Authors:** Dmitry G Deryabin, Ludmila V Efremova, Alexey S Vasilchenko, Evgeniya V Saidakova, Elena A Sizova, Pavel A Troshin, Alexander V Zhilenkov, Ekaterina E Khakina

**Affiliations:** Department of Microbiology, Orenburg State University, Orenburg, Russia; Institute of Cellular and Intracellular Symbiosis, RAS, Orenburg, Russia; Institute for Ecology and Genetics of Microorganisms, RAS, Perm, Russia; All-Russia Research Institute of Beef Cattle Breeding, RAS, Orenburg, Russia; Institute for Problems of Chemical Physics of RAS, Chernogolovka, Russia

**Keywords:** [60]fullerene derivatives, Aqueous suspension, Particle size, Zeta potential, *Escherichia coli*, *Photobacterium phosphoreum*, Bioluminescence inhibition assay, Atomic force microscopy, Toxicity

## Abstract

**Background:**

The cause–effect relationships between physicochemical properties of amphiphilic [60]fullerene derivatives and their toxicity against bacterial cells have not yet been clarified. In this study, we report how the differences in the chemical structure of organic addends in 10 originally synthesized penta-substituted [60]fullerene derivatives modulate their zeta potential and aggregate’s size in salt-free and salt-added aqueous suspensions as well as how these physicochemical characteristics affect the bioenergetics of freshwater *Escherichia coli* and marine *Photobacterium phosphoreum* bacteria. Dynamic light scattering, laser Doppler micro-electrophoresis, agarose gel electrophoresis, atomic force microscopy, and bioluminescence inhibition assay were used to characterize the fullerene aggregation behavior in aqueous solution and their interaction with the bacterial cell surface, following zeta potential changes and toxic effects.

**Results:**

Dynamic light scattering results indicated the formation of self-assembled [60]fullerene aggregates in aqueous suspensions. The measurement of the zeta potential of the particles revealed that they have different surface charges. The relationship between these physicochemical characteristics was presented as an exponential regression that correctly described the dependence of the aggregate’s size of penta-substituted [60]fullerene derivatives in salt-free aqueous suspension from zeta potential value. The prevalence of DLVO-related effects was shown in salt-added aqueous suspension that decreased zeta potential values and affected the aggregation of [60]fullerene derivatives expressed differently for individual compounds. A bioluminescence inhibition assay demonstrated that the toxic effect of [60]fullerene derivatives against *E. coli* cells was strictly determined by their positive zeta potential charge value being weakened against *P. phosphoreum* cells in an aquatic system of high salinity. Atomic force microscopy data suggested that the activity of positively charged [60]fullerene derivatives against bacterial cells required their direct interaction. The following zeta potential inversion on the bacterial cells surface was observed as an early stage of toxicity mechanism that violates the membrane-associated energetic functions.

**Conclusions:**

The novel data about interrelations between physicochemical parameters and toxic properties of amphiphilic [60]fullerene derivatives make possible predicting their behavior in aquatic environment and their activity against bacterial cells.

## Background

[60]fullerene (“buckyball”) is a hollow spherical molecule exclusively composed of 60 carbon atoms. Thirty years after discovery of the fullerenes [1], they are still a topic of interest for potential application, especially with the advent of nanobiology and nanomedicine [2]. Due to unique characteristics, modified forms of [60]fullerene have been intensively investigated for specific biological activities positioned in various fields, from diagnostics [3, 4] to drug delivery [5, 6].

A key condition of the biomedical application of fullerenes is good solubility in aqueous media which is necessary for their administration and distribution in the living systems. Because the pristine buckyball is non-polar, the covalent addition of ionic functional groups is a way of making the fullerene derivatives more hydrophilic than the original molecule [7]. If the additive groups are not localized diffusely but concentrated at one pole of the [60]fullerene core, such chemical functionalization leads to amphiphilic compounds called “amphifullerenes” [8]. As a result, these derivatives contain both hydrophobic and hydrophilic moieties and are self-assembled in aqueous environment to spherical aggregates referred to as “buckysomes” [9]. Although the principal methodologies to prepare aqueous suspensions of [60]fullerene derivatives have been developed, the optimal fine structure of additive groups such as final physicochemical characteristics required for effective solubilization are still not clear. According to the Poisson–Boltzmann theory, this involves the zeta potential—electric difference in the interfacial double layer around dispersed aggregates that plays an important role in the dispersivity and stability of colloidal solutions [10].

The toxicity of [60]fullerene derivatives for live systems (from bacteria to human beings) is probably triggered by their interactions with cell surface, and therefore different dimensions of fullerene aggregates in aqueous suspensions as well as their physicochemical properties can affect the biological response. In particular, the size, surface chemistry, and surface charge were identified as the properties of [60]fullerene derivatives influencing their toxicity against bacterial cells [11, 12]. In vitro studies on anionic carboxyfullerene reported its antibacterial activity on Gram-positive species [13], and in vivo it can protect mice from lethal infection of *Streptococcus pyogenes* [14]. Some aminofullerenes were active against Gram-positive (*Enterococcus faecalis*) and Gram-negative (*Escherichia coli*) bacteria [15]. In the latter reports, the novel cationic [60]fullerene derivatives were tested against *E. coli* cells and have shown a potent antibacterial activity comparable to traditional antibiotics. Therefore, some of these compounds can be positioned as new promising agent for disinfection of waters and soil [16] or as potential antibacterial drugs [17]. However, fundamental cause–effect relationships between the physicochemical properties of [60]fullerene derivatives and their affinity to cell surface in order to better predict their toxicity against bacteria have not yet been clarified.

In the present study, we investigated the effects of the chemical structure of organic addends in 10 originally synthesized amphifullerenes on the aggregate’s size and zeta potential in aqueous suspensions and revealed important correlations between these physicochemical characteristics and contacts of [60]fullerene derivatives with the bacterial cell surface involved in bioenergetics violation and toxic effect.

## Results and discussion

### Characterization of penta-substituted [60]fullerene derivatives in aqueous suspensions

Ten originally synthesized penta-substituted [60]fullerene derivatives used in this study are presented in Table 1. Each compound consists of a hydrophobic “buckyball” cage and five ionic functional groups attached at one pole, thus including amphiphilic properties and significantly increasing solubility in polar media.

**Table 1:**
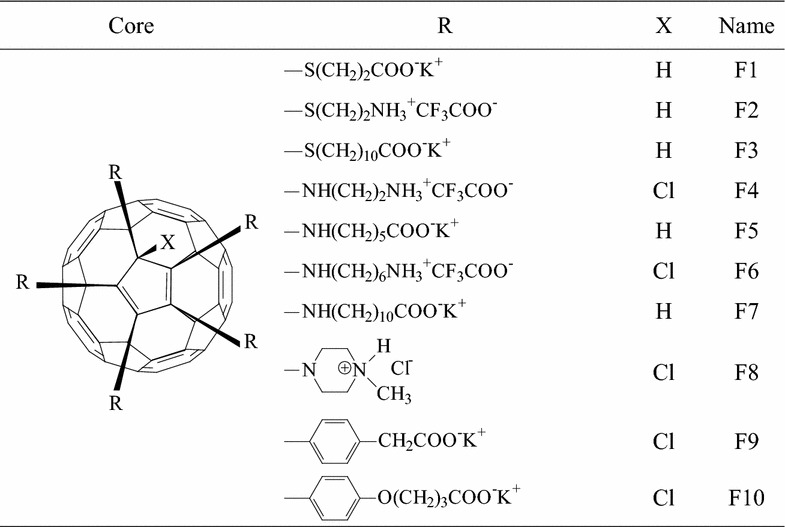
The structural formulas of penta-substituted [60]fullerene derivatives used in this study

**Fig. 1:**
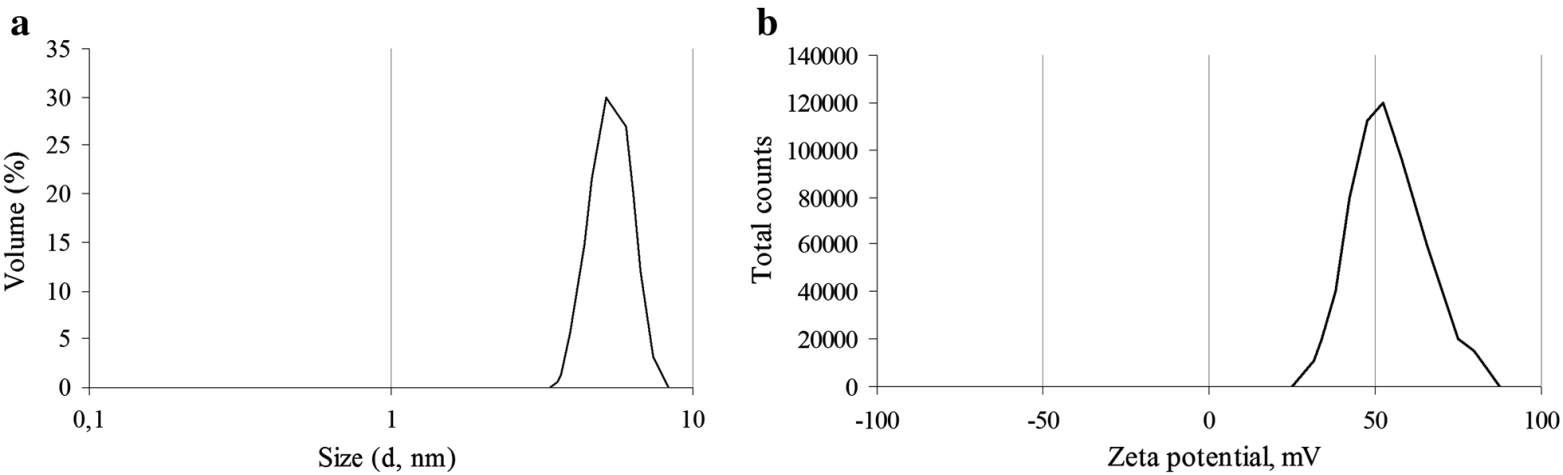
Example of size and zeta potential distribution in the aqueous suspension of F8 penta-substituted [60]fullerene derivative. **a** The diameter of F8 aggregates is 5.2 ± 0.9 nm; **b** the zeta potential value is +52.2 ± 10.6 mV.

The [60]fullerene derivatives formed red-brown colored aqueous suspensions which were used for hydrodynamic size (diameter) measurement when 10 µL aliquots were transferred into the polystyrene cuvettes pre-filled with 1 mL of deionized water. The following light scattering showed nine out of 10 suspensions as a narrow disperse colloidal systems with one well defined peak including 95.5–100% of the particles by volume. However, the hydrodynamic size of individual [60]fullerene derivatives varied over the wide range. The smallest average diameter was determined for F9 (1.9 ± 0.5 nm) and F10 (2.5 ± 0.7 nm) derivatives bearing the residues of the aromatic ring-containing carboxylic acids, while the particles of F5 and F7 derivatives bearing the aminoacid-based addends were somewhat larger (3.1 ± 2.0 nm and 4.6 ± 0.8, respectively). These data also showed alkyl chain length in smallest structure of similar additive groups as the cause of decreasing of [60]fullerene derivatives dispersivity: the particle size of F10 compound was more than F9, and the F7 more than F5. The F4 and F8 compounds bearing with diamine-based functional groups showed particles size less than 10 nm also (Fig. 1a), which, as well as F5, F7, F9, and F10, may represent clusters composed of several fullerene monomers. The hydrodynamic size of F3 particles was established at 10–100 nm diapasons as 26.6 ± 3.9 nm monodisperse aqueous suspension. On the other hand, the average diameter of the aggregates formed by sulfur-containing [60]fullerene derivatives turned out to be more than 100 nm. So, the light scattering data indicated that particles with an average diameter of around 263.4 ± 120.0 and 338.9 ± 118.7 nm predominated in F1 and F2 aqueous suspensions, respectively, suggesting the presence of supramolecular aggregates formed by billions of fullerene monomers. Finally, F6 derivative bearing with diamine) addend produced a polydisperse suspension with two maxima at 22.9 ± 3.0 and 341.2 ± 49.2 nm (65.2 and 34.8% particles by volume, respectively).

Atomic force microscopy (data not shown) revealed that particles of [60]fullerene derivatives exhibit spherical shape and confirmed the hydrodynamic size distribution determined by light scattering according to the Smoluchovski equation.

Zeta potential measurements, evaluated by electrophoretic mobility of colloidal particles of [60]fullerene derivatives, indicated that F1, F3, F5, F7, F9, and F10 compounds acquired negative surface charge from −38.7 ± 6.5 to −57.2 ± 9.1 mV. On the other hand, four compounds show positive surface charge: +43.4 ± 6.4 mV for F2, +49.3 ± 9.8 mV for F4, +41.1 ± 4.5 mV for F6, and +52.2 ± 10.6 mV for F8 (Fig. 1b), respectively.

### Dependence of the particles size in aqueous suspensions of [60]fullerene derivatives on the zeta potential values

In the current experiment, the functionalization variety, as well as precise determination of particle size and zeta potential values were the basis for analysis of the dependence between these physicochemical characteristics of penta-substituted [60]fullerene derivatives, related to their self-organization in aqueous suspension. From a theoretical viewpoint, the zeta potential values suggest that colloidal particles composed of [60]fullerene derivatives functionalized with ionic groups were surrounded by a stable hydrophilic shell of hydrogen-bonded water molecules, which promoted a negative or positive surface charge and prevented the interaction with similar neighboring clusters and aggregates, the zeta potential values were positioned as independent variables (*x*, nm), and the particle size values as dependent variables (*y*, mV).

**Fig. 2:**
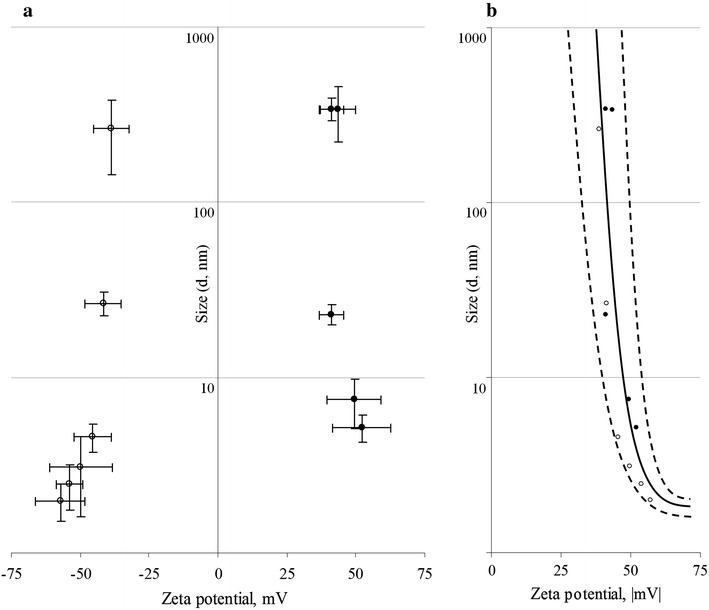
The graph illustrates distribution of particles size and zeta potential values of [60]fullerene derivatives in aqueous suspension. Data analysis with (**a**) and without (**b**) the negative and positive sign of the particles’ surface charge. *White circles* negatively charged compounds; *black circles* positively charged compounds.

Figure 2a demonstrates a symmetrical point’s distribution against the ordinate axis, while the left and right parts of the graph showed a tendency to decrease in particle size with the increasing surface charge. Assuming that negative or positive electric charge is equally important in colloidal systems and that only the average charge value is significant, the diagram was pre-formed in Fig. 2b where the abscissa axis contained the modules of zeta potential values. The following statistical process for estimating the relationships among (*x, y*) variables gave an exponential regression:

$$\hat{y} = \exp (a_{0} + a_{1} \times x),$$

where coefficient a_1_ = −0.2789 and constant a_0_ = 15.7011. This model most correctly described the relationships between physicochemical characteristics of penta-substituted [60]fullerene derivatives in water suspension (the accuracy of the mathematical model characterized by F-criterion is 25.2958; *P* < 0.01). The determination coefficient value for this model, *R*^2^ = 0.7193, led to strict dependence of fullerene aggregation in aqueous suspension from the zeta potential value, determining about three quarters of particle size variability.

The use of the model to predict the clue to zeta potential values required for effective fullerene solubilization and aqueous colloidal suspensions dispersivity yielded two important conclusions. First, this regression showed |39.8| mV as a critical “border” zeta potential value; exceeding this led to self-assembled fullerene aggregates less than 100 nm in diameter. The reported satisfactory zeta potential value agrees with the previously published data for colloid [60]fullerenes in water environment [18, 19] although there are a few more them, probably because of the non-symmetrical distribution of surface charge in the amphifullerene monomer. Second, the predicted zeta potential value required for single-molecular fullerene aqueous suspension having a hydrodynamic particle size of 1 nm was >|56.3| mV. However, the fine structure of investigated amphiphilic compounds containing an expressed hydrophobic pole (“buckyball” cage) makes this perspective unlikely, limiting penta-substituted [60]fullerene derivatives to form clusters composed of more than one fullerene monomer in polar media.

### The zeta potential changes and aggregation behavior of [60]fullerene derivatives in salt-added solution

The environment varies by temperature, pH, ionic strength and dissolved organic matter that alter the behavior of [60]fullerene in natural aquatic systems [20]. According to the classic Derjaguin-Landau-Verwey-Overbeek (DLVO) theory of colloidal stability, the important factor is the presence of electrolytes that varied greatly from freshwater to the marine environment [21]. For this reason, the next step of our experiments was the comparison of [60]fullerene derivative suspensions in salt-free and salt-added media that checked the impact of zeta potential values on aggregation behavior.

Because high electrolyte concentrations limited the laser Doppler micro-electrophoresis, the zeta potential of [60]fullerene derivatives was evaluated by semi-quantitative agarose gel electrophoresis technique [22] with and without 2% NaCl in buffer media while the hydrodynamic size of [60]fullerene derivatives in salt-added suspensions was still measured using non-invasive backscatter (NIBS) optical technology.

**Fig. 3:**
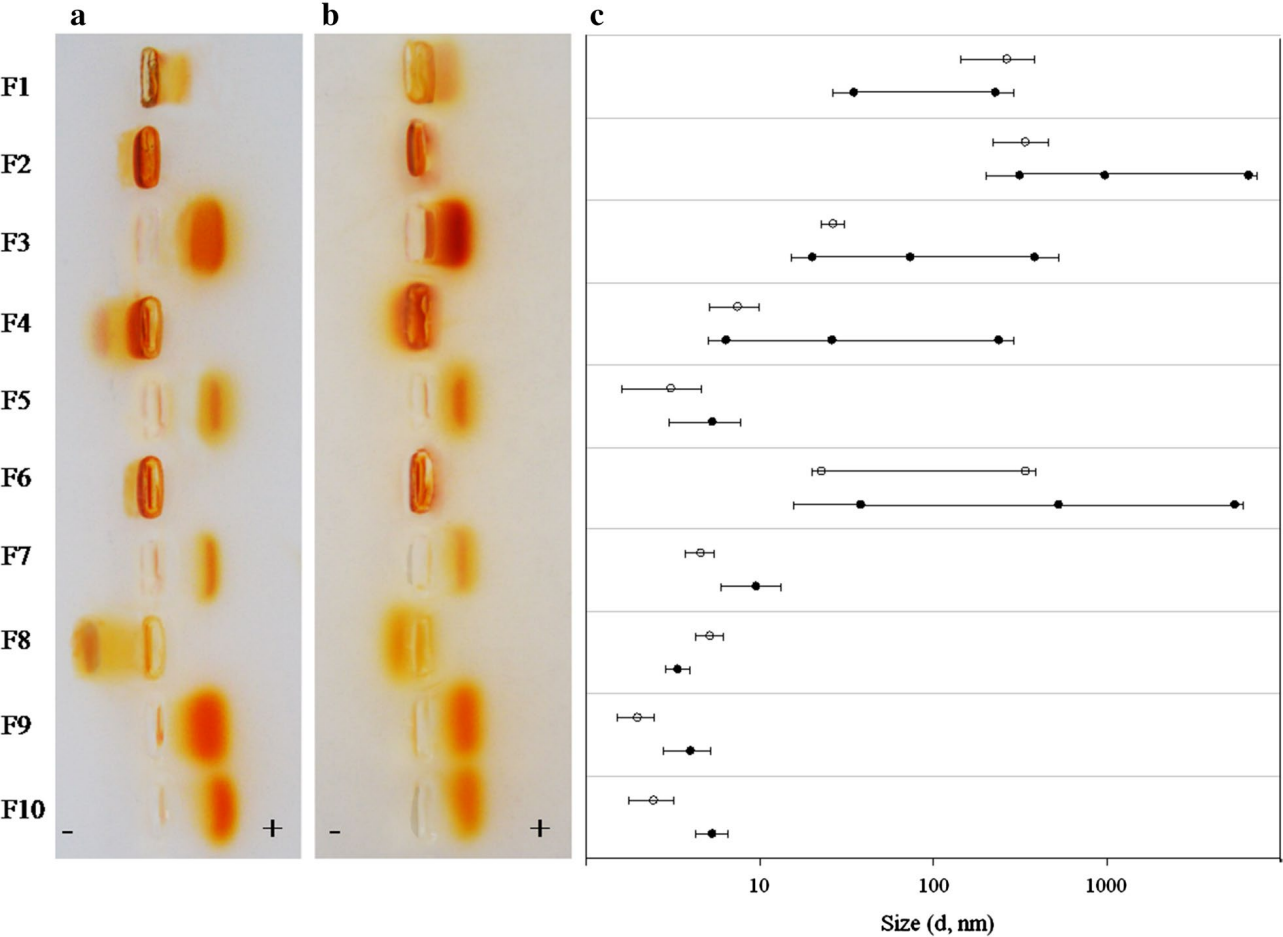
Electrophoretic mobility and aggregation behavior of [60]fullerene derivatives in salt-free and salt-added (2% NaCl) systems. Electroforetic mobility data of F1–F10 compounds in salt-free (**a**) and salt-added (**b**) agarose gel show zeta potential neutralization by high electrolyte concentration. The diameter and polydispersivity parameters (**c**) show the aggregation behavior of F1–F10 compounds in salt-added suspension (*black circles*) in comparison with salt-free suspension (*white circles*).

The electrophoretic mobility of [60]fullerene derivatives in salt-free agarose gel confirmed F1, F3, F5, F7, F9 and F10 compounds as negative surface charged particles and F2, F4, F6 and F8 as positively charged (Fig. 3a). In the presence of 2% NaCl, F2 and F6 lost their electrophoretic mobility whereas other compounds decreased it 1.5–2.5-fold (Fig. 3b), which showed significant neutralization of zeta potential most likely due to opposite charged ions interacting with an electrochemical double layer around the fullerene particles.

A high electrolyte concentration reduced the electrostatic energy barrier between the identically charged nanoparticles, thus leading to an increase in [60]fullerene derivative aggregation expressed differently for individual compounds (Fig. 3c). Being so well dispersed in salt-free media, the F5, F7, F8, F9 and F10 compounds were relatively resistant to electrolyte-induced aggregation while remaining within the range of 10 nm. In turn, F1, F3 and F4 suspensions in the presence of 2% NaCl were polidispersive, while F2 and F6 aggregated dramatically, which is evident in the increase in hydrodynamic size (diameter) of 40.7 and 21.3% of particles over 1000 nm, which in the current experimental context may be interpreted as approaching or exceeding critical coagulation concentrations. On the other hand, in F1 and F8 salt-added suspension we observed partial disaggregation of small particles due to the Debye screening effect [23].

A major implication of this finding is that the behavior of [60]fullerene derivatives in the presence of electrolytes is subjected of several forces distorting the regression dependence as described above. However, the prevalence of the DLVO-type interactions retains the basic dependence of particle size variability from zeta potential value and predicts increased aggregation rates and low bioavailability in aquatic systems of high salinity.

### Effect of penta-substituted [60]fullerene derivatives on bacterial bioluminescence

The toxic effect of [60]fullerene derivatives against freshwater *E. coli* and marine *Photobacterium phosphoreum* strains was evaluated in salt-free and salt-added media, respectively, by measuring the bacterial bioluminescence level as the direct manifestation of the energetic state of integral cells.

**Fig. 4:**
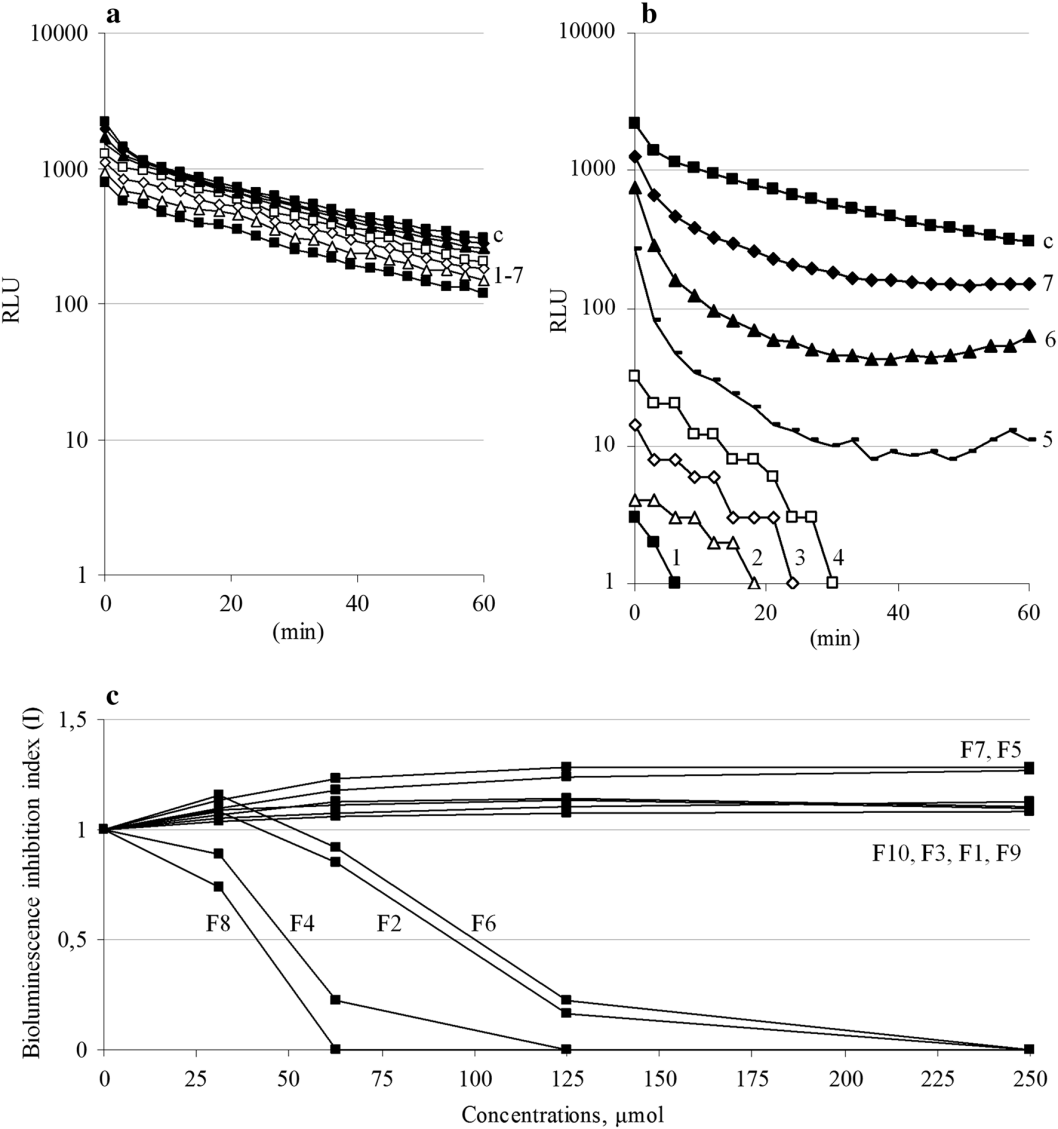
The toxicity of [60]fullerene derivatives evaluated with bioluminescence inhibition assay. The examples of *E. coli* K12 TG1 *lac::luxCDABE* luminescence time course during contact with aqueous suspensions of F10 (**a**) and F8 (**b**) compounds, and the dose–response curves described the changes in the bioluminescence inhibition indexes caused by differing content of penta-substituted [60]fullerene derivatives’ (**c**). Designations (**a**, **b**): ordinate—bioluminescence, RLU; abscissa—time measurement, min. Compounds concentrations: *1* 2000 μmol; *2* 1000 μmol; *3* 500 μmol; *4* 250 μmol; *5* 125 μmol; *6* 62.5 μmol; *7* 31.25 μmol; *c* control. Designations (**c**): ordinate—bioluminescence inhibition index, *I*; abscissa—[60]fullerene derivatives concentrations, μmol.

The universal early event of *E. coli* K12 TG1 *lac::luxCDABE* contact with fullerene suspensions was the rapid light reduction in the first second of instrument registration, which was similar for each [60]fullerene derivative dependent on the compound concentration in the sample. In our experience [24], this effect was not associated with toxicity but was determined by optical distortion of light emission in colored fullerene suspensions. On the other hand, the continued measurement revealed two variants of following bioluminescence kinetics, the first of which had no further changes in light intensity (Fig. 4a), whereas the second show progressive-in-time bioluminescence inhibition (Fig. 4b). According to these comparative data the [60]fullerene derivatives were divided into two groups: (1) non-toxic compounds F1, F3, F5, F7, F9, and F10, which did not affect bacterial energetic metabolism; and (2) toxic compounds F2, F4, F6, and F8, which exerted a concentration-dependent and time-dependent bioluminescence inhibition. Based on final indexes the response curves were compiled (Fig. 4c) and the EC50 toxicological parameters were calculated as 94.3 ± 2.9 μmol for F2, 49.5 ± 1.3 μmol for F4, 99.8 ± 2.7 μmol for F6, and 41.2 ± 1.6 μmol for F8, respectively.

The toxicity of [60]fullerene derivatives against the marine bacterium *P. phosphoreum* B17-677F was determined in 2% NaCl media. Based on the calculated EC50 value, the ranking order for these compounds is as follows: F8 (21.9 ± 0.6 μmol) > F4 (142.7 ± 5.5 μmol) > F6 (234.0 ± 9.7 μmol); whereas the F2 compound at any of the test concentrations with either of the endpoints became inactive in this biotest. Thus, [60]fullerene derivatives, except for the F8 compound, in a salt-added system decreased or lost bioactivity while F1, F3, F5, F7, F9 and F10 were still nontoxic.

Since several penta-substituted [60]fullerene derivatives showed toxic effect against *E. coli* and *P. phosphoreum* cells, the statistical relationship between this bioactivity and fullerenes’ physicochemical properties in salt-free and salt-added aqueous suspension was analyzed. The Pearson correlation coefficient for EC50 toxicological parameters and particles size or zeta potential values was calculated, and the results of random variables were shown as the numerical value and linear dependences for sets of data.

Despite the particle size in aqueous suspension being considered significant for pristine [60]fullerene and fullerenol bioactivity [25, 26], the role of this parameter in penta-substituted [60]fullerene derivatives for *E. coli* K12 TG1 *lac::luxCDABE* and *P. phosphoreum* B17-677F bioluminescence inhibition was insignificant. The correlation coefficients between these variables were *r* = −0.326 and *r* = −0.035 (*P* > 0.05), respectively, and could not be linearized correctly. On the other hand, the correlation between the zeta potential values of the compared [60]fullerene derivatives and toxicity against *E. coli* was very strong, *r* = −0.993 (*P* < 0.01), and could be presented as a linear relationship between the displayed physicochemical and bioactivity parameters. In turn, the presence of a high electrolyte concentration weakened the toxicity of [60]fullerene derivatives against *P. phosphoreum*; nonetheless, this bioactivity was displayed for positively charged compounds only.

Thus, the principal implication of this result is that [60]fullerene derivative toxicity against bacterial cells is strictly determined by positive surface charge whereas no significant toxicity was found for any negatively charged compounds. In addition, the selective analysis of the group of cationic compounds suggested that their bioactivity variability depends on both particle size and zeta potential values, weakened by the high electrolyte concentrations because of charge neutralization and particle aggregation effects.

### AFM evaluation of penta-substituted [60]fullerene derivatives and *E. coli* cells interaction

**Fig. 5:**
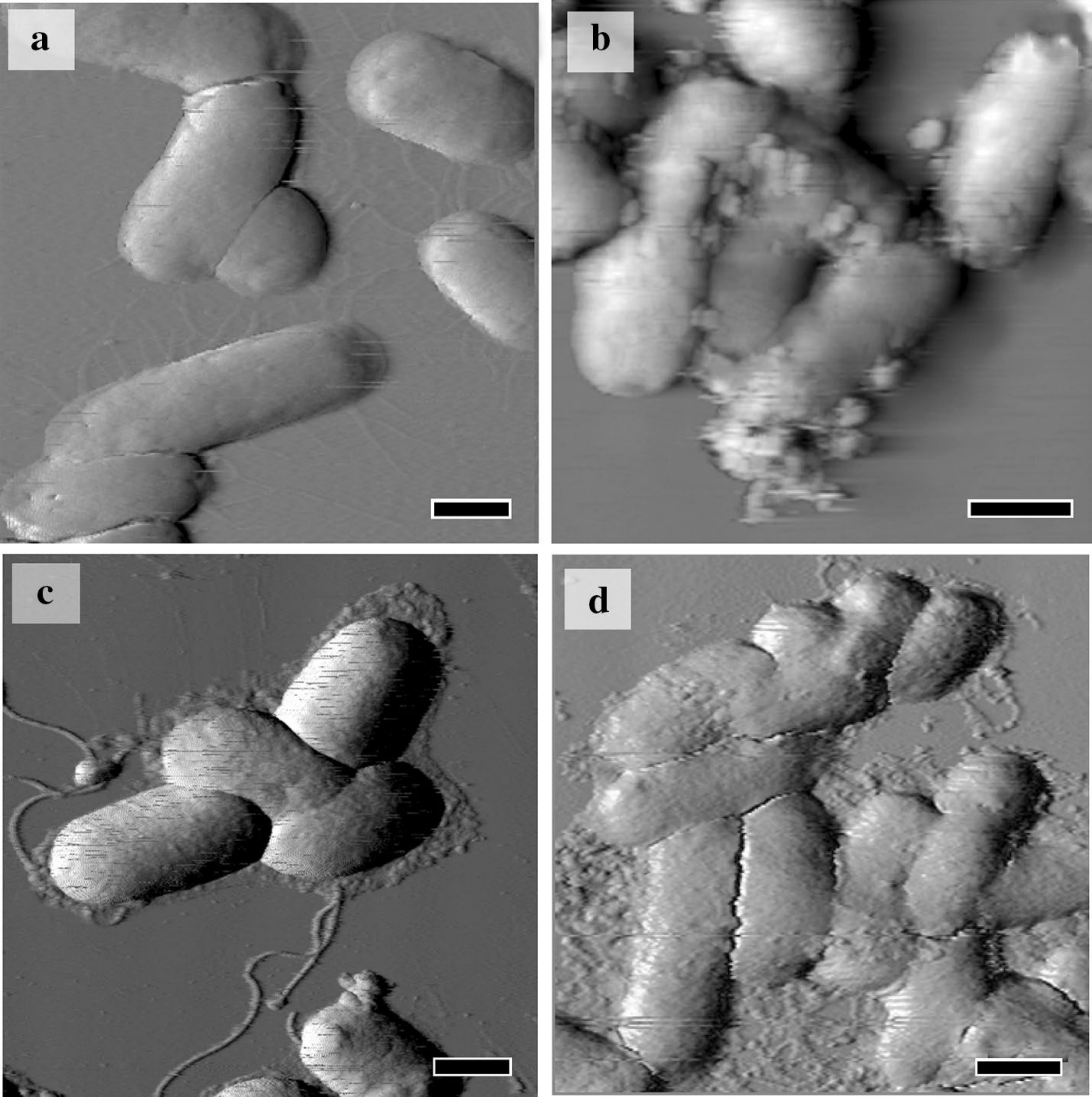
Contact of positively charged [60]fullerene derivatives with bacterial cell surface evaluated with AFM technique. AFM images of *Escherichia coli* cells in control sample (**a**) and treated with F6 (**b**), F4 (**c**) and F8 (**d**) penta-substituted [60]fullerene derivatives. The *scale bar* 1 μm.

Previous studies have shown that pristine [60]fullerene and fullerene derivatives can attach to bacteria and cells [27, 28], but it is not clear whether direct contact is necessary for the antibacterial action. For this reason, the next experiments included the AFM microscopy of intact *E. coli* cells (Fig. 5a) in comparison with the samples treated with non-toxic and toxic penta-substituted [60]fullerene derivatives.

This study did not reveal any bacterial cell surface contacts with negatively charged F1, F3, F5, F7, F9, and F10 compounds, despite various sized round-shaped particles on the mica surface around the cells have being visualized. These features might be attributed to the aggregates of the [60]fullerene derivatives revealed by light scattering measurements (see above). No differences in cell shape, size, or surface roughness were detected when bacterial cells were treated with those compounds which did not exhibit toxic properties (data not shown).

In contrast, the AFM investigation of *E. coli* cells treated with positively charged [60]fullerene derivatives, which exerted a bioluminescence inhibition effect, revealed an affected cell surface structure. The treatment of bacteria with F2 and F6 [60]fullerene derivatives led to covering of bacterial cells with numerous round-shaped particles. For example, treatment of the bacterial cells with F6 led to their covering with multiple aggregates whose sizes varied from 30 to 375 nm with an average size of 296 ± 105 nm (Fig. 5b). On the other hand, in the case of F4 and F8 compounds, the cell surface of the treated bacteria was covered by finely dispersed particles (Fig. 5c, d, respectively) with an average diameter less than 10–20 nm.

In the current experimental context, it is important that the size of the individual round-shaped particles revealed by AFM corresponds well to the light scattering data (see above), thus proving that these clusters represent self-assembled fullerene aggregates interacting with the bacterial cell surface. Other morphological characteristics (length, width, and height) of *E. coli* cells were weak changed only except for F2 compound, where the cell’s morphology changed more significantly. However, no evidences for appearance of grooves or lesions of the outer membrane or intracellular content efflux were observed thus suggesting that membrane disruption can hardly be considered as a leading mechanism of the antibacterial action of the fullerene derivatives [12, 29].

The obtained results demonstrate that the activity of the positively charged penta-substituted [60]fullerene derivatives on *E. coli* cells requires their direct interaction at the initial stage which might affect also bacterial energetic state.

### Effect of penta-substituted [60]fullerene derivatives on zeta potential of *Escherichia coli* cells

**Fig. 6:**
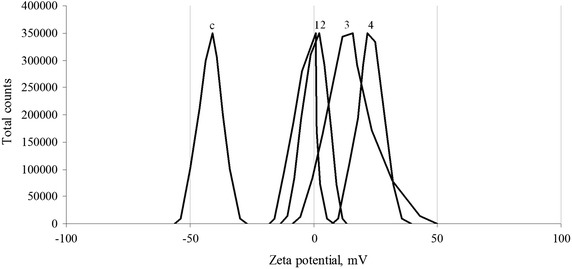
Influence of positively charged [60]fullerene derivatives on zeta potential of *Escherichia coli* K12 TG1 cells. The zeta potential distribution for intact (c) and treated with toxic concentrations of F6 (1), F2 (2), F8 (3), and F4 (4) bacterial suspensions.

We have shown that only the positively charged [60]fullerene derivatives interact with the bacterial cell surface. Therefore, we assumed that electrostatic (Colomb) attraction plays a major role in the antibacterial action of these fullerene derivatives. The experimental zeta potential of *E. coli* K12 TG1 *lac::luxCDABE* cells measured in an aqueous suspension (Fig. 6) corresponds to the surface potential of −40.9 ± 5.8 mV, that fits well to the previously reported data on the charge state of the outer surface of the pro- and eukaryotic cells [30].

The effect of toxic concentrations for penta-substituted [60]fullerene derivatives on the zeta potential of *E. coli* cells was evaluated by laser Doppler micro-electrophoresis when 10 μL aliquots of previously prepared bacteria and fullerene mixtures were added to a cuvette filled with 1 ml of deionized water. It has been shown that introduction of the cationic fullerene derivatives results in significant changes of the zeta potential of *E. coli* cells. Gradually increasing concentrations of fullerenes led to inversion of the surface charge (Fig. 6). For the same concentration of positively charged [60]fullerene derivatives, the effects exerted by F8 and F4 were more severe than those exerted by F2 and F6. The zeta potential of the bacterial cells changed from −40.9 ± 5.8 mV in the control sample to +15.9 ± 10.4 mV (F8) and +21.7 ± 4.9 mV (F4) against +2.3 ± 4.5 mV (F2) and +0.7 ± 3.8 mV (F6). These results are in a good agreement with the data on the antibacterial action of the fullerene derivatives studied using bioluminescence inhibition assay (see above).

In the current experimental context, this inversion was determined by coverage of bacterial surface with positively charged fullerenes. According to the AFM data, the aggregates of the cationic fullerene derivatives had strong Coulombic attraction to the negatively charged *E. coli* cells surface, whereas negatively charged compounds cannot interact effectively with bacteria due to the Coulombic repulsion. Another interesting fact is that comparable concentrations of F4 and F8 compounds with high surface charge and represented by small clusters composed of several fullerene monomers led to more pronounced zeta potential inversion. Most likely, it is due to a complete bacterial surface coverage, while the F2 and F6 aggregates induced less pronounced effect because of the point interaction with the bacterial cell surface.

These results indicate that direct contact of positively charged penta-substituted [60]fullerene derivatives with *E. coli* cells surface is governed mostly by the electrostatic forces, while the following zeta potential inversion may be an early stage of the toxicity mechanism that probably also involves membrane-associated energetic and transport functions [27, 31].

## Conclusions

In this study, we identified the interrelations between basic physicochemical characteristics of 10 originally synthesized penta-substituted [60]fullerene derivatives with amphiphilic properties and their behavior in aqueous suspensions that is significant for toxic effect against freshwater *E. coli* and marine *P. phosphoreum* cells. The obtained results confirmed the Poisson-Boltzmann theory for self-assembly of amphifullerene suspensions. A clear relationship between the zeta potentials of the functionalized [60]fullerene aggregates and their size in salt-free aqueous media has been revealed for the first time. We have also demonstrated a prevalence of Derjaguin-Landau-Verwey-Overbeek-related effects in salt-added aqueous media that decreased zeta potential and affected the aggregation behavior of tested compounds. Notably, the positive surface charge of the fullerene nanoparticles governs their interactions with the negatively charged *E. coli* cells’ surface in a salt-free system and, consequently, their effect on the bacterial bioenergetics, as confirmed by the strong bioluminescence inhibition effect. In turn, the [60]fullerene derivatives’ toxicity against *P. phosphoreum* cells is weakened due to salt-induced zeta potential neutralization and aggregation effects. It has been shown that direct interaction of positively charged [60]fullerene aggregates with bacterial surface and following inversion of the zeta potential of bacterial cells occurs at an early stage of the toxicity mechanism. These results provide novel insights to unveil the molecular mechanisms underlying amphyfullerene biological activity so demonstrating a promising strategy for designing effective antibacterial preparations based on penta-substituted [60]fullerene derivatives.

## Methods

### [60]fullerene derivatives: synthetic procedures and suspension preparation

Ten penta-substituted [60]fullerene derivatives were synthesized according to the previously published highly selective reactions [32, 33] starting from readily available chlorofullerene C_60_Cl_6_ as a precursor. These reactions open up straightforward synthetic routes to many functionalized fullerene derivatives with high yields, for example water-soluble compounds showing various biological activities.

Aqueous suspensions of [60]fullerene derivatives (4 mmol) were prepared in deionized water or 2% NaCl solution in glass vials, vigorously vortexed and sonicated (30 W × dm^−3^, 35 kHz) for 30 min in a water bath. The suspensions were then incubated for about 2 h at 20°C, thus allowing the colloidal systems to reach equilibrium.

### Bacterial strains and culture preparation

An *E. coli* K12 TG1 host-based recombinant strain commercially available as “Ekolum” biosensor (Immunotekh, Russia) was the test object for toxicological screening. This strain carries a pF1 plasmid with the *luxCDABE* operon of marine bioluminescent bacteria *Vibrio fischeri* cloned under the *lac* promoter that gives strong constitutive light emission under standard cultivation conditions. Another sensor strain was the marine bacterium *P. phosphoreum* commercially available as “Microbiosensor B17-677F” [34] carrying the natural *lux* operon in a bacterial chromosome. For both strains the inhibition of bioluminescence is likely due to the toxicity associated with reducing bacterial energetics, because light production requires active NADH and ATP metabolism.

According to the manufacturer’s recommendation, the *E. coli* K12 TG1 *lac::luxCDABE* lyophilized sensor strain was rehydrated with cooled deionized water and *P. phosphoreum* B17-677F with cooled 2% NaCl solution to a concentration about 4 × 10^8^ colony-forming units per 1 mL, exposed for 30 min at 2–4°C and then the temperature was raised to 20 ± 2°C before use, respectively.

### Measurement of the size particles and zeta potential in aqueous suspensions

The size and zeta potential of penta-substituted [60]fullerene derivatives dispersed in salt-free aqueous suspensions were assessed with a laser autocorrelation analyzer, Zetasizer Nano (Malvern Instruments Ltd, United Kingdom).

The basic principle of particle size measurement was a non-invasive back scatter (NIBS) optical technology that estimates real-time changes in the intensity of scattered light as a result of particles undergoing Brownian motion. The samples were placed in 10 × 10 × 45 mm polystyrene cuvettes with four optical faces and optimal transparency along the spectral field from 340–800 nm (Kartell Labware, Italy), and were illuminated by a 633 nm helium–neon laser. The light scattering was measured at an angle of 173° using an avalanche photodiode, and the particles’ size distribution was calculated from the diffusion coefficient according to the Smoluchovski equation, which is known to be rigorously valid for spherical-like particles. The average diameter ± width (nm) of the aggregates of [60]fullerene derivatives in aqueous suspensions was calculated according to the volume size distribution data by using the software of the instrument.

The zeta potential of [60]fullerene derivatives, bacterial cells, and complexes thereof were measured using the technique of laser Doppler micro-electrophoresis. In this method, an electric field is applied across a pair of electrodes to the particle dispersion, which then move with a velocity related to its surface charge values. This parameter is measured using a laser phase analysis light scattering technique (M3-PALS) that enables the calculation of electrophoretic mobility and performs it to mean ± width (mV) zeta potential and zeta potential distribution.

The aggregation behavior of penta-substituted [60]fullerene derivatives dispersed in salt-added aqueous suspensions was analyzed with NIBS optical technology while zeta potential was evaluated with 1% agarose gel electrophoresis with and without 2% NaCl in 0.1% phosphate buffer media (pH 7.2). Electrophoresis was performed in parallel connected chambers at constant voltage of 20 V and current of 200 mA, so that the electric field strength was about 0.7 V/cm. After 30 min of electrophoresis, migration of compounds was evaluated by visible light and the Smoluchowski equation was used to calculate the zeta potential from the electrophoretic mobility.

### Atomic force microscopy

Visualization of investigated compounds, bacterial cells, and contacts between them was performed using an atomic force microscope SMM-2000 (Proton-MIET, Russia) as described previously [35]. Briefly, aliquots (20 µL) of aqueous suspensions of [60]fullerene derivatives, *E. coli* K12 TG1 cells, alone or previously mixed with fullerene suspensions, were applied to freshly prepared mica. The samples were incubated at 93% relative humidity and 20–22°C and scanned in a contact mode using V-shaped silicon nitride cantilevers MSCT-AUNM (Veeco Instruments Inc., USA) with a spring constant of 0.01 N/m and a tip curvature of 10 nm. Quantitative morphometric analysis of the images was performed using the software provided with the instrument.

### Bioluminescent toxicological assay

To assess toxicity of [60]fullerene derivatives against *E. coli* K12 TG1 *lac::luxCDABE* and *P. phosphoreum* B17-677F cells we used a previously described version of bioluminescent analysis for carbon-based nanomaterials [20]. Briefly, aqueous suspensions of [60]fullerene derivatives (4 mmol) were added to the wells of a “Microlite 2+” microplate with non-transparent side walls (Thermo, USA), wherein they were further doubly diluted in sterile deionized water or 2% NaCl solution, from 1:1 to 1:1,024, up to a final volume of 50 μL. Then, 50 μL of a previously prepared suspension of constitutively luminescent *E. coli* or *P. phosphoreum* cells was added to the wells filled with salt-free or salt-added suspensions, respectively. Wells filled with sterile deionized water or 2% NaCl solution and containing an appropriate amount of bacterial biosensor were used as controls.

Bioluminescence measurements were carried out using an LM-01T microplate luminometer (Immunotech, Czech Republic), which dynamically registered the luminescence intensity of the samples for 60 min, estimated in relative light units (RLU). The data were analyzed using KILIA graphing software provided with the instrument. To quantify the bioluminescence inhibition index (I) due to toxicity of [60]fullerene derivatives we used the algorithm $\text{I} = \text{RLU}c0 \times \text{RLU}tn\text{/RLU}cn \times \text{RLU}t0\text{,}$ where *c* and *t* are the RLU values of the control and test samples at the *0*-th and *n*-th minute of measurement. Based on these indexes, we calculated the EC50 toxicological parameters, that is, the concentrations of [60]fullerene derivatives that cause 50% bioluminescence inhibition.

### Statistical analysis

All the experiments were performed using three or more independent series with different preparations. The values were expressed as mean ± standard deviation. The regression analysis for estimating the relationships among zeta potential and particle size values was processed using the software package Statistica V8 (StatSoft Inc., United States). Statistical significance was set at *p* < 0.05.

## Abbreviations

AFM: atomic force microscopy
NIBS: non-invasive back scatter
RLU: relative light units
