# Erratum to: A zeta potential value determines the aggregate’s size of penta-substituted [60]fullerene derivatives in aqueous suspension whereas positive charge is required for toxicity against bacterial cells

**DOI:** 10.1186/s12951-015-0130-4

**Published:** 2015-10-13

**Authors:** Dmitry G. Deryabin, Ludmila V. Efremova, Alexey S. Vasilchenko, Evgeniya V. Saidakova, Elena A. Sizova, Pavel A. Troshin, Alexander V. Zhilenkov, Ekaterina A. Khakina

**Affiliations:** Department of Microbiology, Orenburg State University, Orenburg, Russia; Institute of Cellular and Intracellular Symbiosis, RAS, Orenburg, Russia; Institute for Ecology and Genetics of Microorganisms, RAS, Perm, Russia; All-Russia Research Institute of Beef Cattle Breeding, RAS, Orenburg, Russia; Institute for Problems of Chemical Physics of RAS, Chernogolovka, Russia

## Erratum to: J Nanobiotechnol (2015) 13:50 DOI 10.1186/s12951-015-0112-6

Unfortunately, the original version of this article [1] contained an error. In the author list, Ekaterina A. Khakina was misspelt as Ekaterina E. Khakina. This has now been corrected in the original version.
